# Matrilin-3 Role in Cartilage Development and Osteoarthritis

**DOI:** 10.3390/ijms17040590

**Published:** 2016-04-20

**Authors:** Manjunatha S. Muttigi, Inbo Han, Hun-Kuk Park, Hansoo Park, Soo-Hong Lee

**Affiliations:** 1 School of Integrative Engineering, Chung-Ang University, Seoul 06911, Korea; manjunatha.muttigi@gmail.com; 2 Department of Biomedical Science, CHA University, Seongnam-Si 13488, Korea; 3 Department of Neurosurgery, CHA Bundang Medical Center, CHA University, Seongnam-si 13496, Korea; hanib@cha.ac.kr; 4 Department of Biomedical Engineering, Collage of Medicine, Kyung Hee University, Seoul 02447, Korea; sigmoidus@khu.ac.kr

**Keywords:** matrilin-3, extracellular matrix, cartilage, hypertrophy, interleukin receptor antagonist, osteoarthritis

## Abstract

The extracellular matrix (ECM) of cartilage performs essential functions in differentiation and chondroprogenitor cell maintenance during development and regeneration. Here, we discuss the vital role of matrilin-3, an ECM protein involved in cartilage development and potential osteoarthritis pathomechanisms. As an adaptor protein, matrilin-3 binds to collagen IX to form a filamentous network around cells. Matrilin-3 is an essential component during cartilage development and ossification. In addition, it interacts directly or indirectly with transforming growth factor β (TGF-β), and bone morphogenetic protein 2 (BMP2) eventually regulates chondrocyte proliferation and hypertrophic differentiation. Interestingly, matrilin-3 increases interleukin receptor antagonists (IL-Ra) in chondrocytes, suggesting its role in the suppression of IL-1β-mediated inflammatory action. Matrilin-3 downregulates the expression of matrix-degrading enzymes, such as a disintegrin metalloproteinase with thrombospondin motifs 4 (ADAMTS4) and ADAMTS5, matrix metalloproteinase 13 (MMP13), and collagen X, a hypertrophy marker during development and inflammatory conditions. Matrilin-3 essentially enhances collagen II and aggrecan expression, which are required to maintain the tensile strength and elasticity of cartilage, respectively. Interestingly, despite these attributes, matrilin-3 induces osteoarthritis-associated markers in chondrocytes in a concentration-dependent manner. Existing data provide insights into the critical role of matrilin-3 in inflammation, matrix degradation, and matrix formation in cartilage development and osteoarthritis.

## 1. Introduction

### 1.1. Articular Cartilage and Osteoarthritis

Articular cartilage is a highly specialized tissue covering the end of bones [1]. It mainly consists of hyaline cartilage, with a high collagen II content and a rich proteoglycan matrix [2]. It reduces joint friction by functioning as a shock absorber; thereby protecting the bone ends from mechanical damage [3,4]. Articular cartilage tissue consists of four zones: superficial, intermediate, deep, and calcified cartilage, and has no blood, lymph, or nerve supply [2,3,5]. Cartilage defects can occur owing to osteoarthritis, autoimmune conditions like rheumatoid arthritis, osteochondritis dissecans, and osteonecrosis [6]. However, owing to a lack of blood supply, cartilage repair fails to occur. Furthermore, a dense extracellular matrix (ECM) of cartilage also prevents self-repair by inhibiting the migration of chondrocytes to the injured site [7,8]. Therefore, in this review, we discuss the initiation and progression of osteoarthritis, and the role of matrilin-3 in maintaining cartilage structure, function, and disease development.

Osteoarthritis is a slowly progressive and degenerative disorder characterized by cartilage functional impairment, subchondral bone changes, and osteophyte formation in the absence of autoimmune or anti-inflammatory mechanisms [3,9]. It impairs mobility and lowers extremity function in peripheral joints [9,10,11]. Osteoarthritis is primarily a mechanically induced disease with an etiology to which both acquired and genetic factors contribute [12]. Polymorphisms and mutations in several genes, such as transforming growth factor β *(TGF-*β*)*, bone morphogenetic protein *(BMP)*, growth differentiation factor 5 *(GDF5)*, secreted frizzled-related protein 3 *(FRZB)*, and matrilin-3 *(MATN3)* predisposes individuals to early-onset osteoarthritis [13,14,15,16,17,18,19,20].

In the knee joint, the epiphysis is covered with an epiphyseal plate, also called the growth plate. The epiphyseal plate is composed of resting, proliferative, and hypertrophic zones, which contain reserve/resting, proliferative, and hypertrophic chondrocytes, respectively [21]. During development, long bones elongate via the endochondral ossification process. This process involves chondrocyte terminal differentiation, during which apoptosis occurs. Subsequently, these terminally differentiated cells undergo apoptosis and are replaced by osteoblasts and osteoclasts [22]. Both cartilage and subchondral bone structure and remodeling are regulated by several factors. However, a change in the balance of articular cartilage and subchondral bone remodeling is associated with the initiation and progression of osteoarthritis [23,24,25,26].

### 1.2. Changes in the Remodeling Balance of Cartilage

During normal conditions, articular chondrocytes and subchondral osteoblasts receive mechanical loads and strain on a daily basis and homeostatic mechanisms react accordingly [27]. Eventually, due to predisposing osteoarthritis factors, homeostasis cannot sufficiently compensate for the mechanical load and strain on the body [27]. During this initial period, there is increased proliferation and enhanced remodeling at the cellular level in both cartilage and bone [28]. This is evidently an effort to maintain structural integrity as well as homeostasis in the bone and cartilage. However, an imbalance between chondrocyte anabolism and catabolism leads to the secretion of pro-inflammatory cytokines, including interleukin-1β (IL-1β), matrix metalloproteinases (MMPs), and a disintegrin and metalloproteinase with thrombospondin motifs (ADAMTS) [29,30]. Inflammation in the microenvironment created by IL-1 leads to the loss of ECM components and structure associated with chondrocyte hypertrophy and terminal differentiation. During this process of disease development, chondrocytes express vascular endothelial growth factor (VEGF), Runt-related transcription factor (RUNX2), collagen X, and MMP13 [10,30]. This shift towards hypertrophy is accompanied by the calcification of the ECM around hypertrophic chondrocytes. Hypertrophic changes and increased cellular activity lead to subchondral bone sclerosis, along with a thickening of the cortical plate and remodeling of trabeculae [31].

Several mechanisms are strongly involved in cellular signaling to stimulate vascular invasion by angiogenic factors, such as VEGF. Subsequently, the formation of vascular communication channels in subchondral bone pores facilitates molecular transport between bone and cartilage [32,33,34]. Nevertheless, the chronological order of structural changes in cartilage and subchondral bone during the initiation and progression of osteoarthritis remains unclear [35,36]. It is evident that changes in both cartilage and bone occur before the onset of clinical symptoms. Although cartilage and bone are affected during the initiation and progression of osteoarthritis, their close physical relationship plays a critical role in its repair via biochemical and molecular cross-talk [2,27]. Therefore, a number of studies have focused on the role of growth factors and cytokines in cartilage and bone as targets for the development of new treatments. One potential target is matrilin-3; its role in articular cartilage is discussed below.

## 2. Role of Matrilin-3

### 2.1. Matrilin-3: Bone-Cartilage ECM Modulator

**Figure 1:**
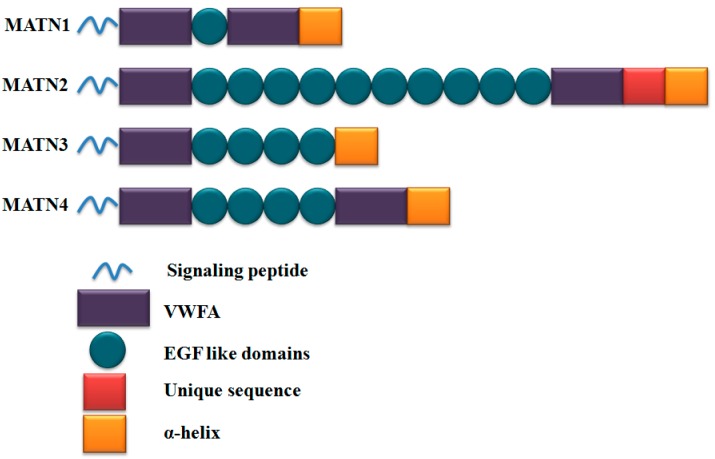
Domain structure of each member of the matrilin family. Each member of the family consists of VWFA, EGF-like domains, and an α-helical oligomerization domain. Abbreviation: MATN, matrilin; VWFA, von Willebrand factor A; EGF, epidermal growth factor.

Matrilin-3 is a non-collagenous ECM protein that functions as an adaptor protein [37,38]. The matrilins form a four-member family, including matrilin-1, matrilin-2, matrilin-3, and matrilin-4. Each member of the family presents one or two Von Willebrand factor A (VWFA) domains, a variable number of epidermal growth factor (EGF)-like domains, and an α-helical coiled-coil domain (Figure 1). Among these, matrilin-3 is the shortest member of the family, containing one von VWFA, four EGF-like domains, and a C-terminal coiled-coil domain [37,38,39,40].

The matrilin-3 gene is expressed in chondroblasts and osteoblasts, but not in hypertrophic chondrocytes [41,42]. Matrilin-3 protein distribution is observed in cartilaginous tissues, such as articular and epiphyseal cartilage, the sternum, and in the cartilaginous anlage of developing bones [41]. It is also present inside both the interterritorial matrix and lacunae of the resting, proliferating, hypertrophic, and calcified cartilage zones and in the perichondrium [41]. As an ECM protein, matrilin-3 plays a critical role in the formation of a filamentous network: collagen (collagen IX)-dependent network connecting cells and the collagen-independent pericellular network [38,43]. Additionally, it is an important component in skeletal development, e.g., it is involved in mesenchymal differentiation, chondrocyte terminal differentiation, de-differentiation, and bone mineral density maintenance [42,44,45,46,47,48,49].

### 2.2. Role of Matrilin-3 in Chondrogenesis, Terminal Differentiation of Chondrocytes, and Ossification

The growth plate or epiphyseal plate contains three types of chondrocytes: reserve/resting, proliferative, and hypertrophic. These three types of chondrocytes are formed under a tightly regulated process of proliferation and terminal differentiation. The hypertrophic chondrocytes undergo apoptosis, degradation, and are then replaced by bone (ossification). Any change in the regulation of this process leads to osteochondral dysplasia or skeletal disorders [21].

Missense mutations in the VWFA domain of matrilin-3 have been observed in patients with multiple epiphyseal dysplasia (MED) [45,50]. MED is characterized by delayed and irregular ossification of the epiphysis. Point mutations in mouse matrilin-3, such as R116W and C299S, increase GADD153 levels, affecting protein folding and trafficking since matrilin-3 is retained and accumulates in the endoplasmic reticulum [51,52]. This results in the reduced formation of the filamentous network around cells. These two mutations (R116W and C299S) are similar to human matrilin-3 point mutations linked to chondrodysplasia [51]. Furthermore, Cotteril *et al.* observed that mutated matrilin-3 is retained in the dilated cisternae of the endoplasmic reticulum [53]. Recently, Jayasurya *et al.* showed that matrilin-3 is essential for TGF-β signaling [54]. In spondyloepimetaphyseal dysplasia (SEMD) and multiple epiphyseal dysplasia (MED), mutations in the matrilin-3 gene cause an aberrant response towards TGF-β and differentiation of ATDC5 chondroprogenitor cells. Furthermore, wild-type matrilin-3 overexpression in ATDC5 chondroprogenitor cells leads to spontaneous chondrogenic differentiation, which is confirmed by increases in collagen II and aggrecan gene expression and in the glycosaminoglycan (GAG) content. Moreover, MED and SEMD mutations cause increased collagen X expression, resulting in premature hypertrophy [54]. In addition, the roles of matrilin-3 in chondrogenesis, premature chondrocyte maturation, and ossification have been confirmed using functional matrilin-3 knockout mice. In matrilin-3 null mice, chondrocytes present in cartilage prematurely transform into a prehypertrophic and hypertrophic phenotype, and form an expanded zone of hypertrophy [48]. BMP2 is a well-known factor responsible for the hypertrophic differentiation of chondrocytes [55]. Yang *et al.* [56] showed that matrilin-3 binds to BMP2 based on a solid-phase binding assay and surface plasmon resonance assay. Binding between matrilin-3 and BMP2 was also confirmed using an immunoprecipitation assay. Yang *et al.* [56] also suggested that matrilin-3 binds to BMP2 and limits its availability to bind the BMP receptor. It was proposed that multiple EGF domains of matrilin-3 cluster together with the coiled-coil domain to form a pocket and bind to BMP-2, thereby inhibiting BMP-2 activity. The binding of matrilin-3 with BMP2 prevents BMP receptor-mediated Smad1 phosphorylation and downstream collagen X expression in chondrocytes [56]. Therefore, matrilin-3 acts as an antagonist to prevent the hypertrophic terminal differentiation of chondrocytes.

### 2.3. Role of Matrilin-3 in Osteoarthritis

**Figure 2:**
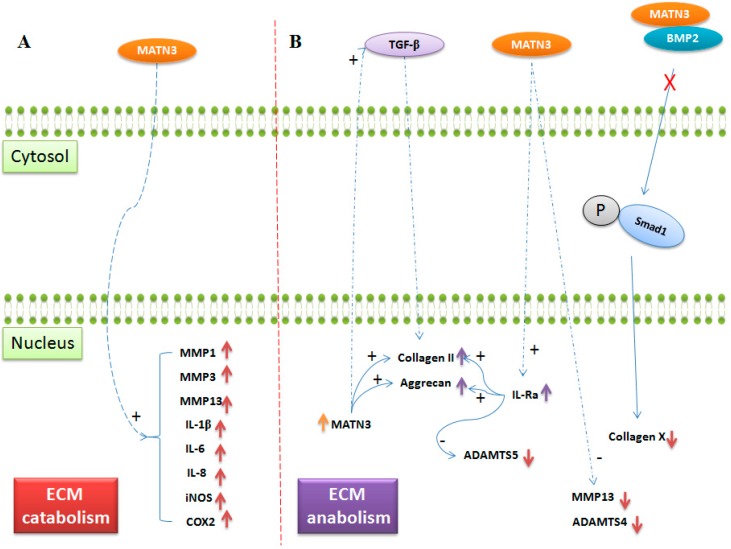
The schematic diagram illustrates the role of matrilin-3 in ECM modulation of cartilage. (**A**) ECM catabolism: Matrilin-3 at supra-physiological concentrations (~5 to 50μg/mL) increases MMP1, MMP3, MMP13, IL-1β, IL-6, IL-8, iNOS, and COX2 expression in chondrocytes (red upward arrows); (**B**) ECM anabolism and hypertrophy: Matrilin-3 mediates the TGF-β signaling pathway. The overexpression of matrilin-3 (orange upward arrow) increases collagen II and aggrecan expression. Matrilin-3 at low concentrations (~100 to 200 ng/mL) increases IL-Ra expression, which increases collagen II and aggrecan expression (purple upward arrows), and reduces ADAMTS5. Recombinant matrilin-3 reduces ADAMTS4 and MMP13 expression (red downward arrows). Matrilin-3 binds to BMP2, thereby inhibiting BMP downstream signaling, collagen X expression, and chondrocyte hypertrophic differentiation (red cross in **B**). Abbreviations: ECM, extracellular matrix; MMP, matrix metalloproteinase; IL, interleukin; iNOS, induced nitric oxide synthase; COX2, cyclooxygenase; TGF-β, transforming growth factor β; IL-Ra, Interleukin receptor antagonist; ADAMTS, a disintegrin and metalloproteinase with thrombospondin motifs; BMP, bone morphogenetic protein. Dashed arrows (blue), unidentified signaling pathways; solid arrow (blue), identified signaling pathway; (+), positive regulatory effect; (−), negative regulatory effect.

Mutations in matrilin-3 are associated with disorders in skeletal development and predisposes individuals to develop osteoarthritis [19,20,45,50,57]. Interestingly, the expression of matrilin-3 is increased in osteoarthritis and its expression levels are correlated with disease severity [58]. Both matrilin-3 mRNA and protein expression were detected in the middle and deep cartilage zone and in subchondral bone. Furthermore, increased matrilin-3 mRNA expression was also found in proliferating chondrocytes near the articular joint surface, without prominent protein expression in the ECM [58]. This increased expression may be a cellular response to the osteoarthritic environment. Previously, Jayasurya *et al.* [47] showed that a soluble recombinant matrilin-3 at concentrations of 100 and 200 ng/mL induces an interleukin 1 receptor antagonist (IL-1Ra), even in the presence of interleukin-1β (IL-1β) in chondrocytes. Matrilin-3 increases collagen II and aggrecan, and reduces ADAMTS5, which is mediated by IL-Ra. Matrilin-3 also suppresses MMP-13 in chondrocytes-suggesting a role in the suppression of hypertrophy caused by inflammation [47]. However, the mechanism by which matrilin-3 regulates IL-Ra is currently unknown. It is possible that matrilin-3 mediates signals via integrins or other plasma membrane receptors such, as EGF receptors (since matrilin-3 have EGF like domains), in chondrocytes [59]. However, the matrilin-3 functions mediated by these receptors have yet to be determined. In contrast to the above findings, Klatt *et al.* [60] showed that 5 to 50 µg/mL matrilin-3 increases the expression of MMP1, MMP3, MMP13, IL-1β, IL-6, IL-8, iNOS and COX-2 in chondrocytes. Increased expression of MMPs and pro-inflammatory markers could increase ECM catabolism and inflammation. Furthermore, the overexpression of ADAMTS4 and ADAMTS5 increases ECM degradation and elevates the levels of the free form of matrilin-3. It is hypothesized that elevated levels of matrilin-3 could in turn increase the expression of ADAMTS4 and ADAMTS5 via IL-1β [60]. In support of this, Klatt *et al.* [61] showed that VWFA domains present in matrilin-3 are responsible for IL-6 expression in primary human chondrocytes (See Figure 2). Matrilin-3 function shifts from an anabolic to a catabolic effect on ECM components, possibly due to a change from a very low concentration range (100 to 200 ng/mL) to a supra-physiological range (5 to 50 μg/mL). A concentration of 5 to 50 μg/mL, which is higher than physiological levels, increases degenerative protease levels and inflammation in chondrocytes, possibly via negative feedback mechanism [47].

Vincourt *et al.* [62] estimated thatmatrilin-3 in the synovial fluid of osteoarthritic patients ranges from 80 ng/mL to 5.7 µg/mL. Furthermore, they compared the effects of the soluble free form of matrilin-3 and the artificially immobilized matrilin-3 on chondrocytes. The treatment of chondrocytes with soluble matrilin-3 increased ECM catabolism, while treatment with the immobilized protein increased ECM anabolism. Soluble matrilin-3 treatment at concentrations of <10 µg/mL induces collagen II expression. On the other hand, matrilin-3 at concentrations of ≥20 µg/mL increases MMP13 expression at both the mRNA and protein levels. In contrast, artificially immobilized matrilin-3 on a plastic surface binds to chondrocytes via a α5β1 integrin-dependent mechanism. Additionally, TGF-β stimulation of chondrocytes allows the integration of matrilin-3 into the chondrocyte ECM. The binding of integrated matrilin-3 increases AKT phosphorylation and in turn, increases chondrocyte survival and ECM synthesis [62]. The above studies suggest that the initial increase in matrilin-3 possibly initiates a defense mechanism that increases IL-Ra, collagen II, and aggrecan in order to limit inflammation and maintain the tensile and elastic strength of cartilage tissues [47]. However, a continual increase in soluble protein to a supra-physiological level is lethal to chondrocytes [60]. However, even when immobilized at high concentrations (50 µg/mL), matrilin-3 functions as an adaptor protein to promote ECM anabolism. This signifies that the integration of matrilin-3 with matrix components is essential to maintain the cartilage ECM [62]. *In vitro* experiments by Pei *et al.* [48] showed that matrilin-1 and -3 are essential to maintain chondrogenesis of synovial fibroblasts. Matrilin overexpression in synovial fibroblasts enhanced chondrogenesis after an initial 3 days of treatment with TGF-β, and maintained chondrogenesis even after the retrieval of TGF-β.

A functional knockout of matrilin-3 in a mouse model showed premature hypertrophy, chondrocyte clustering, osteophyte formation, fibrillation, and the degradation of the articular cartilage surface. Interestingly, mutations in matrilin-3 lead to MED and SEMD in humans, but the matrilin-3 knockout mouse model showed no skeletal deformity. However, these matrilin-3 null mice exhibit increased susceptibility to osteoarthritis during ageing. In matrilin-3 knockout mice, both the levels of collagen II and aggrecan were reduced compared to wild-type mice. These differences affect the structural integrity of the ECM [49]. In addition, matrilin-3 increases IL-Ra in chondrocytes, which in turn regulates the expression of collagen II, aggrecan, and ADAMTS5 [47]. Consequently, the matrilin-3 deletion in mice causes an imbalance in the maintenance of structural integrity with increased inflammation and degradation of ECM components. As a result, matrilin-3 null mice are predisposed to the initiation and progression of osteoarthritis. Hence, matrilin-3 plays an important role in maintaining the structural integrity of cartilage ECM. In addition, matrilin-3 null mice also show an increased bone mineral density [49]. However, it is not known whether the increased bone mineral density in matrilin-3 null mice is due to increased hypertrophy and ossification of the exposed subchondral plate. Matrilin-3 can regulate bone ECM components as evidenced by its expression in bone under normal conditions and its increased levels during osteoarthritis.

## 3. Conclusions and Future Perspectives

The ECM protein matrilin-3 plays a critical role in chondrogenesis, chondrocyte terminal differentiation, and chondrocyte function. However, it has also been implicated in the induction of osteoarthritis-associated gene expression in chondrocytes. At the molecular level, matrilin-3 interacts with TGF-β and BMP-2 to maintain chondrocytes during development and for the homeostatic maintenance of the ECM of cartilage and bone. Mutation and gene knockout studies clearly indicate that matrilin-3 is an indispensable factor in skeletal development and homeostasis.

It is apparent that matrilin-3 affects and mediates the TGF-β and BMP signaling pathways in chondrocytes. Therefore, further studies are required to understand: (1) matrilin-3 function during chondrogenesis in ECM assembly and ECM–cell communication in mesenchymal stem cells; (2) the involvement of matrilin-3 in vasculogenesis during osteoarthritis; and (3) the contribution of matrilin-3 to autoimmune diseases, such as rheumatoid arthritis.

Protein delivery systems, such as micro- or nanoparticles and biomimetic hydrogels, can be used for targeted applications. Micro- or nanoparticles can be usedasmatrilin-3 controlled release delivery systems, since low concentrations of soluble matrilin-3 induce ECM anabolism. Immobilized matrilin-3 also induces ECM anabolism; therefore, immobilizing matrilin-3 on biopolymers, such as hyaluronan, can be a novel approach. Further characterizing the precise functions of matrilin-3 would allow the development of new therapeutic strategies to facilitate the cartilage tissue repair and regeneration.

## Abbreviations

The following abbreviations are used in this manuscript:

TGF-β	Transforming growth factor β
BMP	Bone morphogenetic protein
GDF5	Growth differentiation factor 5
FRZB	Secreted frizzled-related protein 3
MATN3	Matrilin-3
ECM	Extracellular matrix
IL	Interleukin-1
MMPs	Matrix metalloproteinases
ADAMTS	A disintegrin and metalloproteinase with thrombospondin motifs
VEGF	Vascular endothelial growth factor
RUNX2	Runt-related transcription factor
VWFA	Von Willebrand factor A
EGF	Epidermal growth factor
MED	Multiple epiphyseal dysplasia
SEMD	Spondyloepimetaphyseal dysplasia
